# 

**Published:** 1836-04-01

**Authors:** 


### CONTENTS

OF THE

### MEDICO-CHIRURGICAL REVIEW,

No. XLVIII. APRIL 1, 1836.

[BEING No. VIII. OF A DECENNIAL SERIES.]

### REVIEWS.

I.

A Practical Treatise on Urethritis and Syphilis. By William Henry Judd .. .. 305

II.

PATHOLOGICAL ANATOMY.

1. Outlines of Human Pathology. By Herbert Mayo, F.R.S. &c. .. .. .. 328

2. Illustrations of the Elementary Forms of Disease. By Robert Carswell, M.D. 328

Cachectic Abscess .. .. .. .. .. .. .. .. .. .. .. .. .. .. 337
Fibro-Albuminous Tumor .. .. .. .. .. .. .. .. .. .. .. .. 338
Affections of Aponeuroses .. .. .. .. .. .. .. .. .. .. .. .. 339
Affections of the Skin .. .. .. .. .. .. .. .. .. .. .. .. .. 340
Dr. Carswell on Hypertrophy .. .. .. .. .. .. .. .. .. .. .. 345

III.

1. Lectures on Diseases of the Lungs and Heart. By Thos. Davies, M.D. .. .. 353

2. Traité Clinique des Maladies du Cœur. Par J. Bouillaud, M.D. .. .. .. 353

IV.

On the Wisdom and Goodness of God, as manifested in the Creation, Habits, and Instincts of Animals. By the Rev. Mr. Kirby—(3d and last Article) .. .. .. 358

1. Reptiles .. .. .. .. .. .. .. .. .. .. .. .. .. .. .. 358
2. Birds .. .. .. .. .. .. .. .. .. .. .. .. .. .. .. .. 361
3. Man .. .. .. .. .. .. .. .. .. .. .. .. .. .. .. .. 363

V.

ON THE MANAGEMENT OF FRACTURES OF THE EXTREMITIES.

1. Observations on Fractures. By J. Houston, M.D. &c... .. .. .. .. .. .. 365
2. On the Treatment of Fractures without Splints. By Mr. Radley .. .. .. 365

VI.

A Treatise on the Functional and Structural Changes of the Liver, in the Progress of Disease; and on the Agency of Hepatic Derangements in producing other Disorders; with Cases, Dissections, &c. By W. E. Conwell, M.R.I.A. .. .. .. 378

VII.

BRIDGEWATER TREATISES.

Chemistry, Meteorology, and the Functions of Digestion, considered with reference to Natural Theology. By Dr. Prout .. .. .. .. .. .. .. .. .. .. .. .. 385

VIII.

1. Remarks on the Unity of the Body, as illustrated by some of the more striking Phenomena of Sympathy, &c. By Mr. Macilwain .. .. .. .. .. .. 401

2. Practical Observations on the Action of Morbid Sympathies, as included in the Pathology of certain Diseases. By Dr. Wilson .. .. .. .. .. .. .. 401

IX.

THE GANGLIONIC SYSTEM.

1. Recherches Experimentales, &c.—(Experimental Researches on the Functions of the Ganglionic Nervous Systems, &c.) By J. L. BRACHET, M.D. .. .. .. 414
2. De Nervi Sympathetici Humani Fabrica, Usu, et Morbis, &c.—(An Anatomical, Physiological, and Pathological Essay on the Structure, Use, and Diseases of the Sympathetic Nerve.) By J. F. LOBSTEIN, M.D. .. .. .. .. .. .. .. .. } 414

X.

Illustrations of the Comparative Anatomy of the Nervous System.—*Part the First.* By JOSEPH SWAN.. .. .. .. .. .. .. .. .. .. .. .. .. .. .. .. .. .. 435

XI.

The Equilibrium of Population and Sustenance demonstrated; shewing, on Physiological and Statistical Grounds, the Means of obviating the Fears of the late Mr. Malthus and his Followers. By CHARLES LOUDON, M.D. .. .. .. .. .. .. .. 439

XII.

Further Illustrations of the Ulcerative Process. By C. ASTON KEY, Surgeon .. .. 443

### PERISCOPE.

### PART I.

### Spirit of the English Periodicals, and Notices of English Medical Literature.

1. On Diseases of the Spleen. By Mr. Twining .. .. .. .. .. .. .. .. .. 449
2. Cessation of Pulsation in several large Arteries of the Upper and Lower Extremities. By Mr. Crisp; with Commentaries and Examples, by the Editors .. .. 451
3. On the Functions of the Fœtal Kidney. By Robert Lee, M.D. .. .. .. .. .. 453
4. The Russian Puzzle—" Pericardial Exudation ;" with Commentaries .. .. .. 454
5. Temperature of different Parts of the Body in a State of Disease .. .. .. .. 456
6. Anomalous Case of Lithotomy. By Mr. Lizars .. .. .. .. .. .. .. .. 456
7. St. Thomas's Hospital Reports. By Mr. South.
    1. Report on Dropsy. Dr. Burton .. .. .. .. .. .. .. .. .. 458
    2. Report on Delirium Tremens. Dr. Roots .. .. .. .. .. .. .. .. 459
8. Dr. Graves on Medical Statistics.
    1. Chances of Life .. .. .. .. .. .. .. .. .. .. .. .. .. .. .. 461
    2. Lives of Literary Persons .. .. .. .. .. .. .. .. .. .. .. .. 462
    3. Suicides .. .. .. .. .. .. .. .. .. .. .. .. .. .. .. .. 463
9. On the Medicinal Properties of Creosote. By Dr. Elliotson .. .. .. .. .. 463
    1. Phthisis .. .. .. .. .. .. .. .. .. .. .. .. .. .. .. .. 464
    2. Epilepsy .. .. .. .. .. .. .. .. .. .. .. .. .. .. .. .. 464
    3. Neuralgia .. .. .. .. .. .. .. .. .. .. .. .. .. .. .. 464
    4. Vomiting .. .. .. .. .. .. .. .. .. .. .. .. .. .. .. 465
    5. Diabetes .. .. .. .. .. .. .. .. .. .. .. .. .. .. .. 466
    6. Chronic Glanders .. .. .. .. .. .. .. .. .. .. .. .. .. 466
10. Tubercular Development in the Monkey .. .. .. .. .. .. .. .. .. .. 467
11. Flannel in Hot Climates—Dr. Ferguson .. .. .. .. .. .. .. .. .. .. 467
12. Transcendentalisms .. .. .. .. .. .. .. .. .. .. .. .. .. .. 467
    Pulmonary Exhalation .. .. .. .. .. .. .. .. .. .. .. .. .. 468
13. Pathology of Rheumatism .. .. .. .. .. .. .. .. .. .. .. .. .. 469
14. Pathology of Phlegmatia Dolens.. .. .. .. .. .. .. .. .. .. .. .. 469

15. Singular Case of Stercoraceous Vomiting during a Period of Eighteen Months, with the Appearances on Dissection. By Mr. Dickson .. 470
16. Dr. Bostock on the Chemical Composition of Calcareous Tumors .. 472
17. Suppression of the Urinary and Alvine Excretion for several Years !! .. 475
18. Dr. Bright on Periodical Headaches .. 476
19. Extirpation of an enormous Tumor from the Face. By Richard Tuthill, M.D. .. 476
20. The Nakra—a peculiar Indian Disease .. 478
21. Comparative Pathology .. 478
Palsy in the Horse—Mr. Youatt .. 479
22. New Remedy for Epilepsy. The Crusta Genu Equinæ .. 480
23. Ovarian Dropsy accidentally cured .. 482
24. Loss of Uterus and Appendages soon after Delivery. By Mr. Cook .. 482
25. Remarkable Case of Notencephale. By Dr. Hildreth .. 484
26. Remarks on the Phosphorescence of Organized Bodies. By M. Tiedemann .. 486
27. Cases illustrative of the injurious Effects of administering stimulating Remedies during the inflammatory stage of Gonorrhœa. By Henry Johnson .. 488
1. Inflammation of the Absorbents of the Penis .. 489
2. Abscess in the Cellular Tissue of the Perineum .. 490
3. Inflammation of the Prostate and Neck of the Bladder, with Cases .. 492
28. Advantages of possessing no Advantages—Contemporary Liberality in the Hibernian Mode .. 495

PART II.

### Spirit of the Foreign Periodicals, &c.

1. Perforation of the Stomach, opening outwardly—life preserved for three years .. 497
2. On the Epidemics of Influenza. Grippe .. 498
3. On the Application of Sheet-lead, as an Application to Wounds and Ulcers. By M. R. Parisé .. 501
4. On the Treatment of Erectile Tumors. By Professor Lallemand .. 505
5. On the Influence of Hypertrophy of the Heart on Cerebral Diseases. (Eclectic) .. 507
1. Proximity of the Heart to the Cerebrum, as influencing the Cerebral Functions .. 509
2. Hypertrophy of the Left Ventricle, as inducing Cerebral Congestion, &c. .. 510
3. Excessive Impulsion of the Blood on the Brain, as causing Laceration of the Cerebral Pulp, Dilatation or Rupture of Vessels, &c. .. 510
4. Complication of Hypertrophy, with other Forms of Cardiac Disease .. 510
5. Contraction of Left Ventricular Cavity, as favouring Congestion of the Brain, 511
6. The practical Inferences to be deduced from the above data .. 511
6. On the Influence of Lesions of the Right Ventricle of the Heart on the Pulmonary Circulation. (Eclectic.) .. 513
Cases by Bricheteau and others, in the Hospital Necker .. 515
7. Memoirs of the French Royal Academy of Medicine—Sciential Poverty in the Academy .. 517
1. M. Clot Bey on the Introduction of Air into the Cavities of Blood-vessels .. 518
2. On the Torsion of Arteries .. 518
M. Blandin on the same subject .. 519
3. On the Preservation of Timber from the Dry-rot by the Oxymuriate of Mercury. Report of a Committee .. 521
8. On Spinal Diseases, (not Distortions,) by Dr. Enz. From the German—with numerous Cases .. 523
9. Partial Insanity without Fever .. 530
10. On Secondary Abscesses, (Purulent Depôts.) By Dr. Nasse, of Bonn. (Eclectic Article, with Comments) .. 531
11. Remarks on the Proximate Cause of Gout. By Dr. Wendt, of Breslau .. 540
12. Staphyloma treated by Excision of the Cornea, not followed by Destruction of the Eye. By Dr. Buck, of Lubeck .. 543
13. M. Clot Bey on the Plague of Egypt .. 544

### Clinical Review, and Hospital Reports.

GUY'S HOSPITAL.

1. On Diseased Conditions of the Cerebral Arteries. By Dr. Bright, with Cases and Observations .. .. .. .. .. .. .. .. .. .. .. .. .. .. . .. 545
2. Permanent Contraction of all the Extremities—numerous Cartilaginous Deposits on the Arachnoid of the Spine. By Dr. Bright .. .. .. .. .. .. .. .. .. 547
3. An Essay on the Symptoms and Diagnosis of Pericarditis. By Dr. Hughes. With numerous Cases and Commentaries .. .. .. .. .. .. .. .. .. .. .. .. 547

ST. THOMAS'S HOSPITAL.

4. Two Cases of Wounded Arteries. By Mr. Tyrrell .. .. .. .. .. .. . .. 551
    1. Wound of Brachial Artery in Venesection, with Clinical Remarks .. .. 551
    2. Wound of Radial Artery .. .. .. .. .. .. .. .. .. .. .. .. .. . 552
5. Extraction of a Fragment of Catheter from the Bladder, through the Urethra. By Mr. Tyrrell .. .. .. .. .. .. .. .. .. .. .. .. .. .. .. .. .. 553
6. Retention of Urine, arising from Gonorrhœa .. .. .. .. .. .. .. .. .. 554
7. Simple Fracture of the Leg, followed by Loss of Motion of the Side of the Head, &c. By Mr. Tyrrell—with Observations and other Cases. .. .. .. .. .. .. 554

EDINBURGH ROYAL INFIRMARY.

8. Excision of the Superior Maxillary Bone. Mr. Syme .. .. .. .. .. .. .. 558
9. Encysted Tumor in the Neck, cured by Seton .. .. .. .. .. .. .. .. .. 560
10. Ununited Fracture of the Humerus, cured by Excision of the Ends of the Bone .. 561

NEWRY DISPENSARY.

11. Death from Nitric Acid poured into the Ear. By Dr. Morrison .. .. .. .. .. 562
12. Wound of the Epiglottis .. .. . .. .. .. .. .. .. .. .. .. .. .. .. 563

SIR PATRICK DUN'S HOSPITAL.

13. Pathological Observations on the Brain and Nervous System. By Dr. Law .. .. 563
    Extensive Cerebral Hæmorrhage—Ossific Deposits—long-continued Headache .. 564

WESTMINSTER HOSPITAL.

14. Clinical Notes of Cases treated in the Westminster Hospital. By J. Thurnam, M.R.C.S. Apothecary to the Hospital .. .. .. .. .. .. .. .. .. .. .. 565

15. Medullary Sarcoma of the Bones, with Operation. By Mr. Guthrie .. .. .. 565

16. Fæcal Abscess—peculiar Changes in the Respiratory System . .. .. .. .. 567

17. Extra-uterine Fœtation .. .. .. .. .. .. .. .. .. .. .. .. .. .. .. 569

18. Hemiplegia, with peculiar Phenomena .. .. .. .. .. .. .. .. .. .. .. 569

19. Paralysis from Cervical Caries .. .. .. .. .. .. .. .. .. .. .. .. .. 570

20. Hydrocephalus Acutus following Scarlatina .. .. .. .. .. .. .. .. .. .. 570

21. Hydrocephalus Chronicus, with Paralysis.. .. .. .. .. .. .. .. .. .. 571

22. Mania—Emplastro-endermic Method.. .. .. .. .. .. .. .. . .. .. 571

23. Eczema .. .. .. .. .. .. .. .. .. .. .. .. .. .. .. .. .. .. .. 572

24. Clinical Report on Hydrocele of the Neck. By Bransby Cooper, Esq. (GUY'S HOSP.) 575

25. Clinical Report on Aneurism. By Sir Astley Cooper, Bart. .. .. .. .. .. .. 577

26. First successful Case of Ligature of the Common Carotid. By Sir A. Cooper. .. 579

27. Axillary Aneurism cured by tying the Subclavian. By Sir A. Cooper .. .. .. 580

28. Femoral Aneurism cured by tying the External Iliac .. .. .. .. .. .. .. 582

DREADNOUGHT HOSPITAL SHIP.

29. Supposed Aneurismal Tumor in the Orbit, for which the common Carotid was tied, 584

ST. GEORGE'S HOSPITAL.

30. Sir B. Brodie on the Treatment of Bunions and Corns .. .. .. .. .. .. .. 585

### Miscellanies.

1. Cyclopædia of Anatomy, Part V.—Mr. Quain's Anatomical Plates .. .. .. .. .. 587
2. Reclamation of Dr. Marshall.. .. .. .. .. .. .. .. .. .. .. .. .. .. 587
3. Disease of Heart, Liver, &c. By Dr. Johnson . .. .. .. .. .. .. .. .. 588

MEDICAL POLITICS.

1. Pauper Practice considered .. .. .. .. .. .. . .. .. . .. .. .. .. 591
2. Coroner's Inquests—Remuneration .. .. .. .. .. .. .. .. .. .. .. .. 593
3. Insurance Offices .. .. .. .. .. .. .. .. .. .. .. .. .. .. .. .. .. 594
4. Metropolitan University—Medical Reform .. .. .. .. .. .. .. .. .. .. 596

BIBLIOGRAPHICAL RECORD .. .. .. .. .. .. .. .. .. .. .. .. .. .. .. 603

EXTRA LIMITES.

Lunatic Asylum .. .. .. .. .. .. .. .. .. .. .. .. .. .. .. .. .. .. 605

